# Supplementary material for: Optimized Adaptive Radiotherapy with Individualized Plan Library for Muscle-Invasive Bladder Cancer Using Internal Target Volume Generation
Source: Cancers (Basel). 2022 Sep 26;14(19):4674. doi: 10.3390/cancers14194674 (PMC9564375; doi:10.3390/cancers14194674)
Supplement: Supplementary file 1 [file cancers-14-04674-s001.zip › Supplementary Figure S1.pdf]

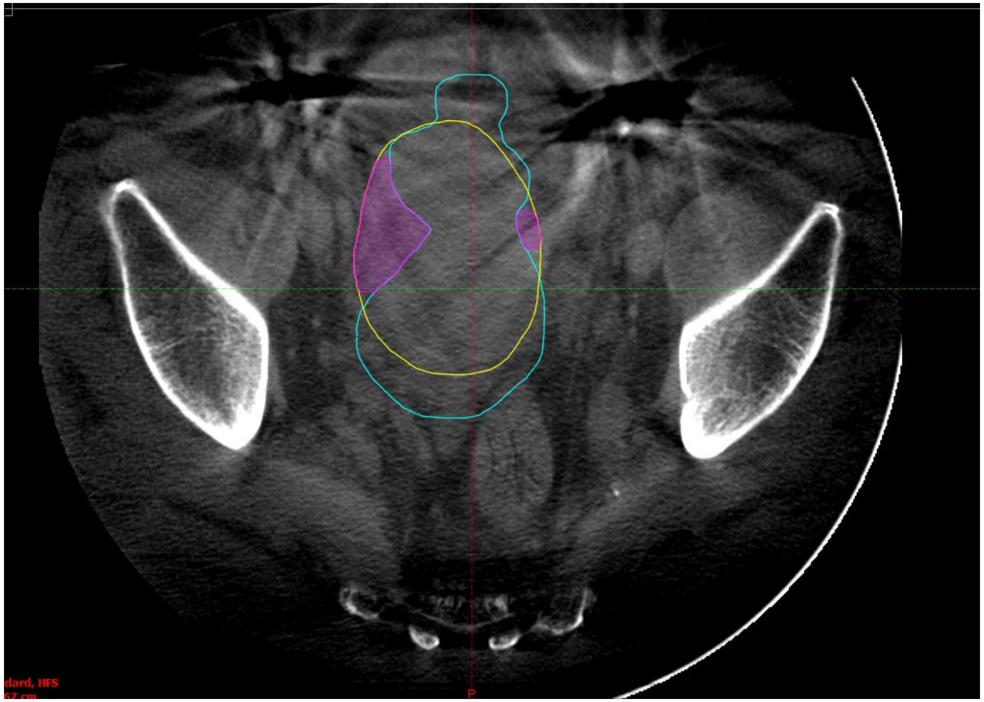

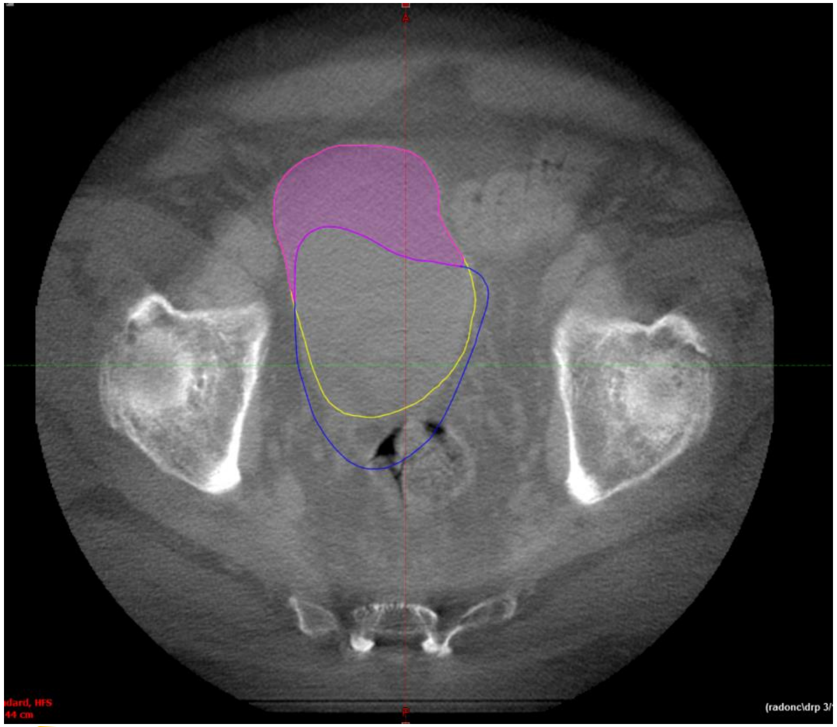

no.	Library	Good	Fair	Poor	Null
1	22	21	0	1	0
2	23	19	3	1	0

postCBCT bladder – PTV original : calculation

If, calculation volume <0.05 : **Fair**

If, calculation volume ≥0.05 : **poor**
