# Supplementary material for: Optimized Adaptive Radiotherapy with Individualized Plan Library for Muscle-Invasive Bladder Cancer Using Internal Target Volume Generation
Source: Cancers (Basel). 2022 Sep 26;14(19):4674. doi: 10.3390/cancers14194674 (PMC9564375; doi:10.3390/cancers14194674)
Supplement: Supplementary file 1 [file cancers-14-04674-s001.zip › Supplementary Tables.pdf]

Supplementary Table S1. post treatment CBCT bladder coverage.

Patient no.	Library count	Good	Fair	Poor	Null
1	22	21	0	1	0
2	23	19	3	1	0
3	22	20	2	0	0
4	22	22	0	0	0
5	22	18	0	4	0
6	21	20	0	0	1
7	20	20	0	0	0
8	21	18	2	1	0
9	21	21	0	0	0
10	19	19	0	0	0
11	22	22	0	0	0
12	22	22	0	0	0
13	22	20	0	2	0
14	22	19	0	3	0
15	20	20	0	0	0
16	22	22	0	0	0
17	22	22	0	0	0
18	21	19	2	0	0
19	22	21	1	0	0
Total	408	385	10	12	1

Supplementary Table S2. Patterns of disease recurrence.

Site of recurrence	n (%)
Local NMIBC	3 (15.8%)
Local MIBC	2 (10.5%)
Local+distant	1 (5.3%)
Distant only	2 (10.5%)
Total	8 (42.1%)

MIBC, muscle-invasive bladder cancer; NMIBC, non-muscle-invasive bladder cancer.

Supplementary Table S3. Univariate analysis and multivariate analysis for 2-year OS.

Variable	Number	Univariate analysis		Multivariate analysis	
		2-yr OS (%)	P-value	HR (95% CI)	P-value
Age (years)					
<80	8	66.7%	0.514	-	
≥80	11	59.7%			
Gender					
Male	13	50.8%	0.173	-	
Female	6	100%			
CCI					
<4	12	53.9%	0.356	-	
≥4	7	83.3%			
ECOG					
0-1	18	62.8%	0.650	-	
2-3	1	100%			
T stage					
2	13	68.8%	0.482	-	
3-4	6	53.3%			
Induction CTx					
Received	6	80.0%	0.507	-	
Not received	13	62.5%			
CCRT					
Received	10	66.7%	0.381	-	
Not received	9	55.6%			

Supplementary Table S4. Organ at risk constraints.

	Constratints	
Recutm	50 Gy	20%
	70 Gy	7%
Small bowel	V35	180cc
	V40	100cc
	V45	65cc
	D max	50 Gy
Femur head	V40	35%
	V45	10%
	Dmax	50 Gy
