# Supplementary figures and images for: Optimized Adaptive Radiotherapy with Individualized Plan Library for Muscle-Invasive Bladder Cancer Using Internal Target Volume Generation
Source: Cancers (Basel). 2022 Sep 26;14(19):4674. doi: 10.3390/cancers14194674 (PMC9564375; doi:10.3390/cancers14194674)

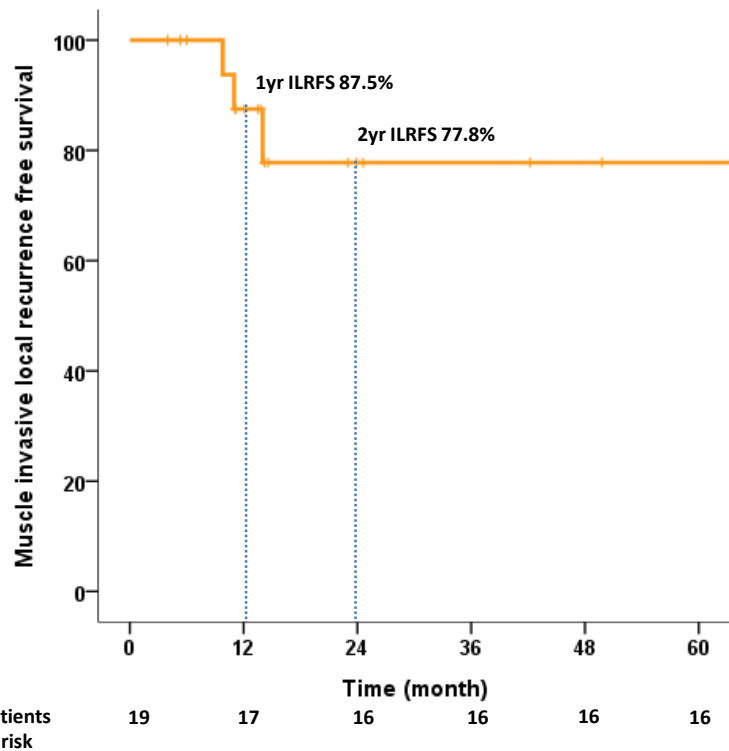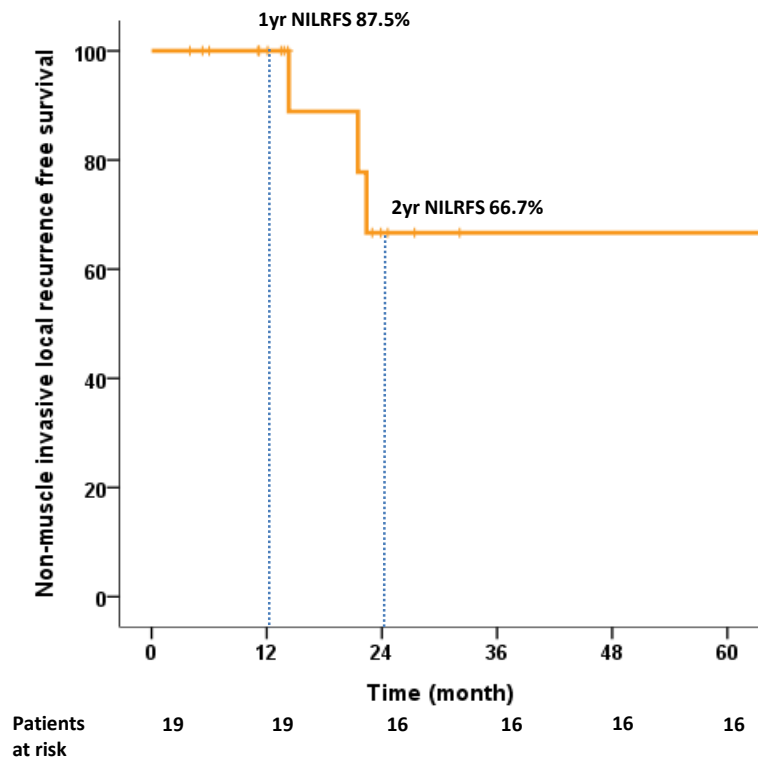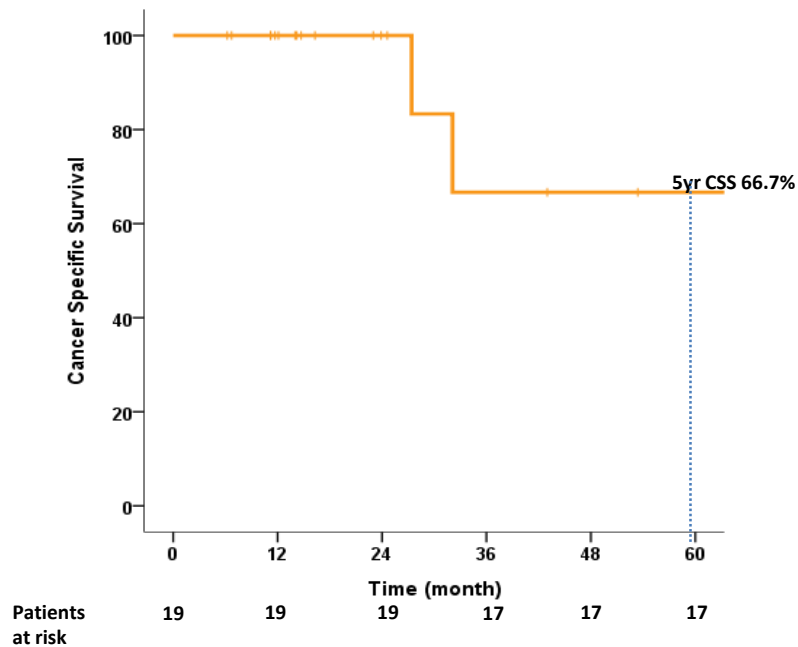

Supplement: Supplementary file 1 [file cancers-14-04674-s001.zip › Supplementary Figure S2.pdf]
